# Supplementary material for: The modified arterial reservoir: An update with consideration of asymptotic pressure (P∞) and zero-flow pressure (Pzf)
Source: Proc Inst Mech Eng H. 2020 May 5;234(11):1288–99. doi: 10.1177/0954411920917557 (PMC7705641; doi:10.1177/0954411920917557)
Supplement: Supplementary_data_1rev – Supplemental material for The modified arterial reservoir: An update with consideration of asymptotic pressure (P∞) and zero-flow pressure (Pzf) [file Supplementary_data_1rev.pdf]

Study name	n	Method	Duration of inflow cessation, s	Species	Comments	Ref
Sagawa 1975	11	Intercept	NA	Dog	Anesthetized, venous pressure set to zero	(1)
Bottomley 1976	12	Stopped flow/intercept	3-4/NA	Rabbit	Anesthetized, bypass. P_{zf} were calculated from stopped flow in some experiments but some values were derived from extrapolation. Both techniques were reported to give similar estimates. Venous pressure was between 0 to 3mmHg (never >7mmHg), but no other quantitative data.	(2)
Jackman 1977	20	Stopped flow	30	Dog	Anesthetized, on bypass. Venous pressure set to zero.	(3)
Samar 1978	30	Stopped flow	12	Rat	Anesthetized. P_{zf} reported to be 10mmHg higher than MCFP which was 7.6±0.7 mmHg in 30 rats - P_{zf} estimated as MCFP +10mmHg. The duration of flow cessation seems to be have been somewhat variable and the value used was that reported in the discussion.	(4)
Ehrlich 1980	6	Stopped flow	~3	Dog	Anesthetized. Manipulated leg data was used. Venous pressure controlled.	(5)
Yamamoto 1980	6	Stopped flow	4-5	Rat	Anesthetized. In some cases the duration of flow cessation was up to 38s but the typical duration is reported here.	(6)
Sylvester 1981	18	Stopped flow	20	Dog	Anesthetized, double bypass.	(7)
Braakman 1983	6	Intercept	NA	Dog	Anesthetized, bypass. Pressures converted from kPa. MCFP not measured	(8)
Brunner 1983	7	Stopped flow	20	Dog	Anesthetized, bypass. Data from when venous outflow prevented (condition 2).	(9)
Ehrlich 1984	11	Intercept	NA	Dog	Anesthetized, vagal arrest. MCFP not measured.	(10)
Shoukas 1984	10	Stopped flow	20	Dog	Anesthetized, bypass. Data from carotid sinus pressure of 125mmHg, stopped flow for 20s. Venous pressure controlled.	(11)

Ogilvie 1990	11	Stopped flow	6-8	Pig	Anesthetized, data following circulatory arrest with acetylcholine used.	(12)
Hiesmayr 1992	4	Stopped flow	10	Human	Anesthetized, bypass. Data presented as difference from CVP estimated from graph. MCFP estimated from gradient assuming similar confidence interval to P_{zf}.	(13)
Brunner 1993	6	Stopped flow	30	Dog	30s arrest of pump, data estimated from bar plot also showing standard deviations. MCFP not measured.	(14)
Urzua 1997	30	Intercept	NA	Human	Anesthetized, on bypass. MCFP not reported	(15)
Urzua 1998	11	Stopped flow	4	Dog	Anesthetized. 4s aortic occlusion. MCFP not reported	(16)
Jellinek 2000	10	Fibrillation	11-28	Human	Anesthetized. Data presented as difference from CVP estimated from graph and text. CVP estimated as ~10mmHg so this was differences. MCFP estimated from right atrial pressure after arrest.	(17)
Schipke 2003	82	Fibrillation	13	Human	Anesthetized. Data derived from 323 fibrillation sequences but number of observations (N) taken as patients not episodes. MCFP estimated from right atrial pressure after arrest. In some cases duration of fibrillation exceeded 13s.	(18)
Kottenberg-Assenmacher 2009	10	Fibrillation	30	Human	Anesthetized. MCFP estimated from central venous pressure after arrest.	(19)
Maas 2012	10	Intercept	NA	Human	Anesthetized. Mechanically ventilated post-cardiac surgery patients	(20)

SUPPLEMENTARY TABLE S1. References identified in systematic review and included in meta-analysis. Abbreviations: CVP – central venous pressure; MCFP – mean circulatory filling pressure; NA – not applicable, P_{zf} – zero flow pressure.

References

1. Sagawa K, Eisner A. Static pressure-flow relation in the total systemic vascular bed of the dog and its modification by the baroreceptor reflex. Circ Res. 1975;36(3):406-13.
2. Bottomley MG, Mainwood GW. A square-pulse flow method for measuring characteristics of the arterial bed. J Appl Physiol. 1976;40(3):425-33.
3. Jackman AP, Green JF. Arterial pressure-flow relationships in the anesthetized dog. Ann Biomed Eng. 1977;5(4):384-94.
4. Samar RE, Coleman TG. Measurement of mean circulatory filling pressure and vascular capacitance in the rat. Am J Physiol. 1978;234(1):H94-100.
5. Ehrlich W, Baer RW, Bellamy RF, Randazzo R. Instantaneous femoral artery pressure-flow relations in supine anesthetized dogs and the effect of unilateral elevation of femoral venous pressure. Circ Res. 1980;47(1):88-98.
6. Yamamoto J, Trippodo NC, Ishise S, Frohlich ED. Total vascular pressure-volume relationship in the conscious rat. Am J Physiol. 1980;238(6):H823-8.
7. Sylvester JT, Gilbert RD, Traystman RJ, Permutt S. Effects of hypoxia on the closing pressure of the canine systemic arterial circulation. Circ Res. 1981;49(4):980--7.
8. Braakman R, Sipkema P, Westerhof N. Steady state and instantaneous pressure-flow relationships: characterisation of the canine abdominal periphery. Cardiovascular research. 1983;17(10):577-88.
9. Brunner MJ, Greene AS, Sagawa K, Shoukas AA. Determinants of systemic zero-flow arterial pressure. Am J Physiol. 1983;245(3):H453--60.
10. Ehrlich W, Baer RW, Paidipaty BB, Randazzo R. Instantaneous renal arterial pressure-flow relations in anesthetized dogs. Am J Physiol. 1984;246(5 Pt 2):H702-9.
11. Shoukas AA, Brunner MJ, Frankle AE, Greene AS, Kallman CH. Carotid sinus baroreceptor reflex control and the role of autoregulation in the systemic and pulmonary arterial pressure-flow relationships of the dog. Circ Res. 1984;54(6):674-82.
12. Ogilvie RI, Zborowska-Sluis D, Tenaschuk B. Measurement of mean circulatory filling pressure and vascular compliance in domestic pigs. Am J Physiol. 1990;258(6 Pt 2):H1925-32.
13. Hiesmayr M, Jellinek H, Perger P, Augustin W, Simon P, Kainz C, et al. Is there a systemic waterfall in humans? Evidence from haemodynamic measurements during artificial circulatory arrest. Journal of Cardiothoracic and Vascular Anesthesia. 1992;6(1):2.

14. Brunner MJ, Bishop GG, Shigemi K, Freeman JP, Chung D. Arterial pressure-flow relationships in hypertensive dogs: effect of carotid sinus baroreflex. Am J Physiol. 1993;265(3 Pt 2):H986-92.
15. Urzua J, Meneses G, Fajardo C, Lema G, Canessa R, Sacco CM, et al. Arterial pressure-flow relationship in patients undergoing cardiopulmonary bypass. Anesth Analg. 1997;84(5):958-63.
16. Urzua J, Nunez G, Meneses G, Lema G. Hemodilution Decreases Critical Closing Pressure But Does Not Increase the Aortic to Distal Pressure Gradient in Dogs. Internet journal of Anesthesiology. 1998;3:1--4.
17. Jellinek H, Krenn H, Oczenski W, Veit F, Schwarz S, Fitzgerald RD. Influence of positive airway pressure on the pressure gradient for venous return in humans. Journal of applied physiology (Bethesda, Md : 1985). 2000;88(3):926-32.
18. Schipke JD, Heusch G, Sanii AP, Gams E, Winter J. Static filling pressure in patients during induced ventricular fibrillation. Am J Physiol Heart Circ Physiol. 2003;285(6):H2510--5.
19. Kottenberg-Assenmacher E, Aleksic I, Eckholt M, Lehmann N, Peters J. Critical closing pressure as the arterial downstream pressure with the heart beating and during circulatory arrest. Anesthesiology. 2009;110(2):370-9.
20. Maas JJ, Pinsky MR, Geerts BF, de Wilde RB, Jansen JR. Estimation of mean systemic filling pressure in postoperative cardiac surgery patients with three methods. Intensive Care Med. 2012;38(9):1452-60.
